# Effects of cardiovascular risk factors and pre-existing diseases on the short-term outcome of Takotsubo syndrome

**DOI:** 10.1007/s00508-024-02326-4

**Published:** 2024-02-06

**Authors:** Roya Anahita Mousavi, Andreas Schober, Christina Kronberger, Emilie Han, Brigitte Litschauer, Gernot Pichler, Roza Badr Eslam

**Affiliations:** 1 Department of Cardiology, Medical University of Vienna, Vienna, Austria; 2 Karl Landsteiner Institute for Cardiovascular and Critical Care Research, Vienna, Austria; 3 Department of Cardiology, Clinic Floridsdorf, Vienna, Austria; 4 Department of Clinical Pharmacology, Medical University of Vienna, Vienna, Austria; 5 Department of Cardiology, Medical University of Vienna, Währinger Gürtel 18–20, 1090 Vienna, Austria

**Keywords:** Mortality, ST-elevated myocardial infarction, Hypertension, Smoking, Psychiatric disorders

## Abstract

**Background:**

The effects of cardiovascular risk factors (CVRF) on the development of most acute cardiac conditions are well established; however, little is known about the frequency and effects of CVRF in Takotsubo syndrome (TTS) patients.

**Objective:**

The aim of our study was to compare the frequency of CVRF and pre-existing diseases (PD) of TTS patients to ST-elevation myocardial infarction (STEMI) patients and analyze their effects on short-term outcome.

**Methods:**

We analyzed the frequency of CVRF (hypertension, hyperlipidemia, type II diabetes mellitus, smoking, chronic kidney disease, family history) as well as somatic and psychiatric PD at admission in TTS patients and compared them with STEMI patients. Their effect on short-term outcome was calculated using a combined endpoint of cardiogenic shock, cardiopulmonary resuscitation, mechanical ventilation, and/or in-hospital death.

**Results:**

In total, 150 TTS and 155 STEMI patients were included in our study. We observed a higher frequency of psychiatric (30% vs. 7%, *p* < 0.001), neurological (5% vs. 0%, *p* = 0.01), and pulmonary (18% vs. 5%, *p* < 0.001) PD in TTS patients as compared to STEMI patients. There were less smokers (47% vs. 61%, *p* = 0.03) and patients with hyperlipidemia (24% vs. 51%, *p* < 0.001) in the TTS cohort than in the STEMI cohort. None of the CVRF or PD behaved as an independent predictor for adverse short-term outcome in TTS patients.

**Conclusion:**

Psychiatric, neurological, and pulmonary pre-existing diseases are more common in TTS than in STEMI patients. Interestingly, PD and CVRF do not seem to have any impact on the short-term outcome of TTS patients.

## Introduction

Takotsubo syndrome (TTS) also known as Takotsubo cardiomyopathy, stress cardiomyopathy or broken heart syndrome, describes an acute form of heart failure with typical left ventricular wall motion abnormalities. The most common symptoms of TTS are acute chest pain, dyspnea, troponin elevation and electrocardiogram (ECG) changes [1], thus, difficult to distinguish from those of acute myocardial infarction (AMI).

Stressful physical (e.g., surgery, seizure) and emotional (e.g., conflicts, death of a family member) situations have been described as trigger factors for TTS although the exact pathophysiologic mechanisms of the development of TTS are incompletely understood [2].

Hypotheses for the pathogenesis of TTS include sympathetic stimulation, coronary microvascular dysfunction, abnormalities of the central autonomic nervous system and inflammation [2, 3].

TTS has been initially categorized as a benign disease; however, recent studies have shown that the mortality of TTS patients is similar to patients with AMI [4, 5]. Physical trigger factors, male sex, atrial fibrillation and acute neurologic and psychiatric disorders have been described as independent predictors for adverse short-term outcome of TTS [5–7]; however, the exact mechanism of these factors leading to increased in-hospital complications are not completely understood.

Hypertension, dyslipidemia, type II diabetes mellitus, smoking, chronic kidney disease and positive family history for cardiovascular disease are commonly known as conventional cardiovascular risk factors facilitating the development and progression of coronary artery disease and are targets of therapeutic management and prevention of AMI and cardiovascular diseases [8]. Evidence whether these cardiovascular risk factors have any effect on the development and prognosis of TTS is still scarce and little is known about the impact of pre-existing diseases on the outcome of TTS.

The aim of our study was to compare the frequency of pre-existing diseases and conventional cardiovascular risk factors of patients with TTS to ST-elevation myocardial infarction (STEMI) patients.

Furthermore, we wanted to analyze the effects of cardiovascular risk factors and pre-existing diseases on the short-term outcome of patients with TTS.

## Methods

Data of consecutive patients with TTS from 2009–2022 were retrospectively collected from the Departments of Cardiology of the Medical University of Vienna and the Clinic Floridsdorf and compared to consecutive patients with STEMI from 2019.

The diagnosis of TTS was made according to the revised Mayo Clinic criteria [9] :(1) transient left ventricular wall motion abnormalities beyond a single epicardial coronary artery distribution territory, (2) absence of obstructive coronary artery disease or acute plaque rupture, which could explain the wall motion abnormalities, (3) evidence of new ECG abnormalities and/or elevation in cardiac troponin levels and (4) absence of myocarditis.

For data collection all clinical records were reviewed and cardiovascular risk factors, medical history, laboratory testing and in-hospital outcome were evaluated.

This study was approved by the Ethics Committee of the Medical University of Vienna and the Ethics Committee of the City of Vienna and was conducted in accordance with the ethical standards outlined in the Declaration of Helsinki.

### Cardiovascular risk factors and pre-existing diseases

The collected cardiovascular risk factors were hypertension, hyperlipidemia, type II diabetes mellitus, smoking, chronic-kidney disease, and positive family history for cardiovascular disease.

Pre-existing diseases were categorized into somatic and psychiatric diseases. Somatic diseases were subcategorized into cardiac (e.g., pre-admission diagnosed heart failure, atrial fibrillation, structural heart diseases), pulmonary (e.g., chronic obstructive pulmonary disease [COPD], asthma), autoimmune (e.g., myasthenia gravis, Takayasu syndrome), neurological (e.g., epilepsy, stroke) and hormonal diseases (e.g., hypothyroidism, hyperparathyroidism).

Psychiatric pre-existing diseases were subcategorized into depression, anxiety disorders and substance abuse disorders.

### Outcome

The collected in-hospital outcome parameters were cardiogenic shock, cardiopulmonary resuscitation (CPR), the use of catecholamines, invasive and noninvasive mechanical ventilation as well as in-hospital death. The data were extracted by review of patient’s medical charts.

Adverse short-term outcome was defined using a combined endpoint of cardiogenic shock, CPR, the use of catecholamines, invasive and non-invasive mechanical ventilation and/or in-hospital death.

### Statistical analysis

Categorical variables were expressed as absolute numbers and percentages and continuous variables were expressed either as mean ± standard deviation or as median and interquartile range dependent on the distribution.

Differences in the frequency of pre-existing diseases and cardiovascular risk factors between TTS and STEMI patients were analyzed with χ^2^-test, Student’s t‑test, or Mann-Whitney U‑test.

Outcome analysis of TTS patients was performed with binary logistic regression model using the combined endpoint of cardiogenic shock, CPR, the use of catecholamines, invasive and non-invasive mechanical ventilation and/or in-hospital death. Variables that had a *p*-value < 0.1 in univariate analysis were included into multivariate binary logistic regression model.

A (two sided) *p*-value of less than 0.05 was defined as statistically significant. Data were managed using MS Excel 2016 (Microsoft, Redmond, CA, USA). All statistical analyses were performed using SPSS statistics Version 27.0 (IBM Corporation, Armonk, NY, USA).

## Results

In total 150 patients with TTS and 155 patients with STEMI were included into the study. All clinical characteristics and laboratory parameters are shown in Table 1.

**Table 1:** Clinical characteristics and laboratory parameters of TTS and STEMI patients

	Takotsubo (*n* = 150)	STEMI (*n* = 155)	*p*-value
*Baseline characteristics*			
Age (years)	66 ± 14	63 ± 13	0.06
Female sex	122 (82%)	41 (27%)	*< 0.001*
BMI	22 ± 6	28 ± 6	*<* *0.001*
Systolic BP admission	134 ± 29	135 ± 30	0.86
Diastolic BP admission	80 ± 18	82 ± 16	0.41
HR admission	84 ± 18	80 ± 23	0.12
Physical trigger	44 (30%)	n.a.	n.a.
Emotional trigger	48 (36%)	n.a.	n.a.
*Cardiovascular risk factors*			
Hypertension	79 (53%)	83 (56%)	0.68
Hyperlipidemia	35 (24%)	75 (51%)	*<* *0.001*
Smoking	56 (47%)	86 (61%)	0.03
Type II diabetes mellitus	16 (11%)	24 (16%)	0.20
Positive family history	23 (19%)	26 (17%)	0.75
Chronic kidney disease	7 (5%)	9 (6%)	0.55
*Pre-existing diseases*			
Somatic diseases	126 (85%)	112 (73%)	*0.01*
Cardiac diseases	40 (27%)	39 (25%)	0.69
Pulmonary diseases	26 (18%)	7 (5%)	*<* *0.001*
Hormonal diseases	24 (16%)	19 (12%)	0.36
Autoimmune diseases	27 (18%)	20 (13%)	0.23
Neurological diseases	7 (5%)	0 (0%)	*0.01*
Psychiatric diseases	44 (30%)	11 (7%)	*<* *0.001*
Depression	30 (20%)	7 (5%)	*<* *0.001*
Anxiety disorders	5 (3%)	0 (0%)	*0.02*
Substance abuse	11 (8%)	3 (2%)	*0.02*
*Symptoms at admission*			
Chest pain	106 (74%)	132 (91%)	*<* *0.001*
Dyspnea	50 (35%)	14 (10%)	*<* *0.001*
Arrythmia	19 (13%)	19 (13%)	0.98
*Laboratory parameters*			
Troponin T admission (ng/L)	245 [69.5–514.5]	152.5 [35.3–1230]	0.97
Troponin T peak (ng/L)	415 [241–770]	2952 [1015.8–5421.5]	*<* *0.001*
Nt-pro-BNP admission (pg/ml)	1461 [365.63–4474.5]	259 [75.2–1439]	*<* *0.001*
NT-pro-BNP peak (pg/ml)	4235.5 [1477.5–10395.5]	1441 [677.3–3492.7]	*<* *0.001*
CKMb admission (U/L)	36.4 [27.83–64.65]	115.5 [50.8–286]	*<* *0.001*
CKMb peak (U/L)	42.5 [31.0–95.5]	134 [69–287]	*<* *0.001*
CK admission (U/L)	143 [87.0–249.5]	294 [125–1125.5]	*<* *0.001*
CK peak (U/L)	224 [110.8–224.0]	1203 [595.5–2247]	*<* *0.001*
CRP admission (mg/dl)	0.53 [0.2–1.8]	0.3 [0.2–0.78]	0.07
CRP peak (mg/dl)	2.5 [0.6–7.4]	1.4 [0.7–5.0]	0.24
WBC admission (G/L)	10.48 [7.8–13.6]	11.7 [9.4–14.2]	*0.002*
WBC peak (G/L)	12 [9.1–14.5]	12.5 [10.2–14.8]	0.26
HDL (mg/dl)	55.5 [44.0–70.5]	39.5 [34–49.3]	*<* *0.001*
LDL (mg/dl)	97 [76.5–117.2]	118.3 [90–148.3]	*<* *0.001*
Hb (g/dl)	13.3 [12.0–14.3]	14.8 [13.4–15.6]	*<* *0.001*
GFR (ml/min/1.73m^2^)	61.0 [42.6–87.8]	80 [64.3–96.8]	*<* *0.001*
Creatinine (mg/dl)	0.8 [0.69–1]	0.9 [0.8–1.1]	*<* *0.001*
TSH (μlU/ml)	1.36 [0.8–2.4]	1.6 [0.9–2.7]	0.25
*Outcome parameters*			
Cardiogenic shock	18 (12%)	16 (10%)	0.64
CPR	16 (12%)	16 (10%)	0.92
Catecholamine use	19 (13%)	13 (9%)	0.22
Invasive mechanical ventilation	23 (15%)	13 (9%)	0.06
In-hospital death	8 (5%)	13 (8%)	0.30

Data are presented in mean ± standard deviation, median [interquartile range], or absolute numbers and percentages

*BMI* bodymass index, *BP* blood pressure, *HR* heart rate,* n.a.* not applicable, *CK* creatine kinase, *CKMb* creatine kinase myocardial band, *NT-pro-BNP* N-terminal pro–B-type natriuretic peptide, *STEMI* ST-elevated myocardial infarction, *CRP* C-reactive protein, *WBC* white blood cell count, *HDL* high density lipoprotein, *LDL* low density lipoprotein, *Hb* hemoglobin, *GFR* glomerular filtration rate, *TSH* thyroid stimulating hormone, *CPR* cardiopulmonary resuscitation

In the TTS group 122 patients (82%) were female with a mean age of 66 ± 14 years, 48 patients (36%) had an emotional trigger factor and 44 (30%) a physical trigger factor. The most common physical trigger factors were pulmonary exacerbation (*n* = 12), postoperative TTS (*n* = 8), falls with or without bone fractures (*n* = 6) and acute neurological disorders (*n* = 4).

There were significantly more women in the TTS group than in the STEMI cohort (82% vs. 27%, *p* < 0.001).

No statistically significant differences between TTS and STEMI patients regarding the frequency of hypertension, type II diabetes mellitus, positive family history for cardiovascular disease or chronic kidney disease were found.

Patients with TTS had a significantly lower body mass index (22 vs. 28, *p* < 0.001), a lower percentage of hyperlipidemia (24% vs. 51%, *p* < 0.001) and were less frequently smokers (47% vs. 61%, *p* = 0.03) than STEMI patients. The frequency of somatic (85% vs. 73%, *p* = 0.01) and psychiatric (30% vs. 7%, *p* < 0.001) pre-existing diseases was higher in the TTS group as compared to STEMI patients.

In the subcategories of somatic diseases TTS patients had significantly more pulmonary (18% vs. 5%, *p* < 0.001) and neurological (5% vs. 0%, *p* = 0.01) pre-existing diseases than STEMI patients. Regarding cardiac, autoimmune, and hormonal comorbidities, no statistically significant differences between the two groups were found as shown in Table 1.

As for subcategories of psychiatric pre-existing diseases more TTS patients suffered from depression (20% vs. 5%, *p* < 0.001), anxiety disorders (3% vs. 0%, *p* = 0.02) and substance abuse disorders (8% vs. 2%, *p* = 0.02) than patients with STEMI.

Of note, patients with TTS had significantly higher admission and peak NT-pro-BNP levels as well as lower peak troponin T, creatine kinase (CK) and CK myocardial band type values than STEMI patients (Table 1).

### Outcome analysis

In total 30 (20%) TTS and 23 (15%) STEMI patients had an adverse in-hospital outcome using the combined endpoint (*p* = 0.23). The observed in-hospital mortality rate was 5% (*n* = 8) in TTS patients and 8% (*n* = 13) in STEMI patients (*p* = 0.3). Furthermore, no statistically significant differences in the frequency of cardiogenic shock, catecholamine use or invasive mechanical ventilation between TTS and STEMI patients were found as shown in Table 1.

Clinical characteristics of patients with adverse short-term outcome are shown in Table 2.

**Table 2:** Clinical characteristics of TTS and STEMI patients with adverse in-hospital outcome

	Adverse in-hospital outcome		
	Takotsubo (*n* = 30)	STEMI (*n* = 23)	*p*-value
*Baseline characteristics*			
Age (years)	65 ± 13	65 ± 14	0.91
Female sex	23 (77%)	6 (26%)	*<* *0.001*
BMI	25 ± 4	29 ± 12	0.05
Systolic BP admission	112 ± 26	105 ± 24	0.36
Diastolic BP admission	68 ± 9	69 ± 18	0.81
HR admission	93 ± 27	106 ± 37	0.30
Physical trigger	15 (52%)	n.a.	n.a.
Emotional trigger	8 (33%)	n.a.	n.a.
*Cardiovascular risk factors*			
Hypertension	16 (53%)	9 (45%)	0.56
Hyperlipidemia	3 (10%)	7 (37%)	*0.02*
Smoking	13 (65%)	9 (56%)	0.59
Type II diabetes mellitus	3 (10%)	6 (29%)	0.09
Positive family history	4 (16%)	2 (11%)	0.60
Chronic kidney disease	3 (10%)	3 (10%)	0.90
*Pre-existing diseases*			
Somatic diseases	24 (80%)	15 (68%)	0.33
Cardiac diseases	7 (23%)	4 (18%)	0.65
Pulmonary diseases	7 (23%)	2 (9%)	0.18
Hormonal diseases	5 (17%)	2 (9%)	0.43
Autoimmune diseases	4 (13%)	3 (14%)	0.98
Neurological diseases	4 (14%)	0 (0%)	0.07
Psychiatric diseases	12 (40%)	4 (18%)	0.09
Depression	5 (17%)	2 (9%)	0.43
Anxiety disorders	2 (7%)	0 (0%)	0.22
Substance abuse	5 (17%)	2 (9%)	0.43
*Symptoms at admission*			
Chest pain	8 (32%)	13 (65%)	*0.03*
Dyspnea	14 (52%)	4 (20%)	*0.03*
Arrythmia	16 (57%)	14 (67%)	0.50
*Laboratory parameters*			
Troponin T admission ng/L	309 [43.75–765.5]	566 [113.5–3108]	0.20
Troponin T peak ng/L	736 [267-1482]	4315 [2645–20674]	*<* *0.001*
Nt-pro-BNP admission pg/ml	2389 [446.4–5866.5]	303.2 [162–5895.8]	0.15
NT-pro-BNP peak pg/ml	6368 [1487–16465]	2742 [716.5–9257.7]	0.24
CKMb admission U/L	41.15 [31.1–73.0]	79 [53–312]	*0.02*
CKMb peak U/L	62.55 [39.7–118.3]	245 [109–397]	*0.001*
CK admission U/L	155 [68.5–482.3]	489 [220.5–2527]	*0.01*
CK peak U/L	375 [106.5–1086.5]	1948 [964–2898.5]	*<* *0.001*
CRP admission mg/dl	0.9 [0.3–2.2]	0.7 [0.2–1.5]	0.43
CRP peak mg/dl	8.7 [3.0–14.8]	10.4 [2.6–15.6]	0.89
WBC admission G/L	11.2 [8.4–16.0]	13.8 [12.0–16.2]	0.06
WBC peak G/L	14.1 [11.8–21.7]	14.2 [13.0–17.6]	0.78
HDL mg/dl	46 [35.5–62]	36 [31.3–44]	0.07
LDL mg/dl	87.6 [72.5–112.4]	93 [71–121]	0.75
Hb g/dl	12.8 [11.8–14.1]	14.9 [13.2–16.5]	*<* *0.001*
GFR ml/min/1.73m2	36 [33.3–45.7]	68 [36.5–92]	0.06
Creatinine mg/dl	0.8 [0.7–1.1]	1.1 [0.9–1.6]	*0.03*
TSH μlU/ml	1.95 [1.1–2.68]	1.8 [0.9–5.4]	0.72

Data presented in mean ± standard deviation, median [interquartile range] or absolute numbers and percentages

*BMI* Body mass index, *BP* blood pressure, *HR* heart rate, *n.a.* not applicable, *CK* creatine kinase, *CKMb* creatine kinase myocardial band, *NT-pro-BNP* N-terminal pro–B-type natriuretic peptide, *STEMI* ST-elevated myocardial infarction, *CRP* C-reactive protein, *WBC* white blood cell count, *HDL* high density lipoprotein, *LDL* high density lipoprotein, *Hb* Hemoglobin, *GFR* glomerular filtration rate, *TSH* thyroid stimulating hormone, *CPR* cardiopulmonary resuscitation

The frequency of hyperlipidemia was significantly lower in TTS patients than in STEMI patients with adverse in-hospital outcome (10% vs. 37%, *p* = 0.02).

Results of binary logistic regression analysis of the TTS cohort are displayed in Table 3.

**Table 3:** Binary logistic regression analysis of TTS patients using the combined endpoint

	Univariate		Multivariate	
	OR	*p*-value	OR	*p*-value
*Baseline Characteristics*				
Age	0.99	0.72	–	
Female sex	0.70	0.47		
BMI	0.99	0.76		
Physical trigger	3.21	*0.01*	4.75	*0.01*
Emotional trigger	0.89	0.80	–	
*Cardiovascular risk factors*				
Hypertension	1.0	1.0	–	
Hyperlipidemia	0.30	0.06	0.51	0.41
Type II diabetes mellitus	0.92	0.90	–	
Smoking	2.36	0.09	1.74	0.36
Chronic kidney disease	3.17	0.15	–	
Positive family history	0.79	0.70		
*Pre-existing diseases*				
Somatic diseases	0.66	0.43	–	
Cardiac diseases	0.77	0.58		
Pulmonary diseases	1.59	0.36		
Autoimmune diseases	0.65	0.46		
Hormonal diseases	1.06	0.91		
Neurological diseases	6.13	*0.02*	8.59	0.07
Psychiatric diseases	1.83	0.16	–	
Depression	0.74	0.58		
Anxiety disorder	2.74	0.28		
Substance abuse	3.73	*0.04*	4.15	0.11

*BMI* body mass index, *OR* odds ratio

In univariate analysis physical trigger factors (odds ratio [OR] = 3.21, *p* = 0.01), neurological pre-existing diseases (OR = 6.13, *p* = 0.02) and history of substance abuse (OR = 3.73, *p* = 0.04) behaved as predictors for adverse in-hospital outcome in TTS patients. Furthermore, there was a trend towards better prognosis in patients with hyperlipidemia (OR = 0.30, *p* = 0.06). In multivariate analysis physical trigger factors were the only independent predictors for adverse short-term outcome in TTS patients (OR = 4.75, *p* = 0.01).

## Discussion

The three main findings of our study are as follows: 1) TTS patients have a higher frequency of somatic and psychiatric pre-existing diseases than STEMI patients, 2) TTS patients have a similar frequency of hypertension, type II diabetes mellitus, chronic kidney disease and positive family history for cardiovascular disease as STEMI patients, and 3) pre-existing diseases and cardiovascular risk factors do not seem to have a significant impact on the short-term outcome of TTS patients.

The high frequency of cardiovascular risk factors in TTS patients has already been described in previous studies [10, 11]; however, to the best of our knowledge, this is the first study to assess the differences in the frequency of pre-existing diseases and all conventional cardiovascular risk factors between TTS and STEMI patients.

We observed a higher frequency of somatic and psychiatric pre-existing diseases in TTS patients as compared to STEMI patients. The high frequency of psychiatric disorders observed in our study has also been described in previous studies [5, 10] and psychiatric disorders are postulated to have a pathogenic role in TTS [2]. In the subcategories of somatic diseases only pulmonary and neurological diseases were more frequent in the TTS group. Acute neurological disorders as well as acute pulmonary disorders have been described as physical trigger factors for TTS [2] which may explain the higher frequency of those pre-existing diseases in the TTS cohort. Interestingly, we observed no differences in the frequency of pre-existing cardiac, autoimmune, and hormonal diseases between TTS and STEMI patients. High levels of catecholamines triggered by major physical illness or sudden stress are thought to be involved in the development of TTS [3]. Our data support the hypothesis by revealing an increased frequency only in pre-existing disease subcategories that are known to act as physical trigger factors in TTS patients in contrast to those with STEMI.

### Cardiovascular risk factors

Hypertension, hyperlipidemia, smoking, type II diabetes mellitus, chronic kidney disease, and positive family history for cardiovascular disease are commonly known risk factors for the development of coronary artery disease. Therefore, a higher frequency of those risk factors would be expected in the STEMI cohort.

Interestingly, in our study cohort no statistically significant differences in the frequency of hypertension, type II diabetes mellitus, chronic kidney disease and positive family history for cardiovascular disease between TTS and STEMI patients were found.

One hypothesis for the pathophysiology of TTS is microvascular dysfunction [3, 12, 13], which has been shown to be present in all included TTS patients of a recent study by Sans-Roselló et al. [14].

Previous studies described associations between the development of microvascular dysfunction and hypertension [15], type II diabetes mellitus [16], chronic kidney disease [17], smoking [18] as well as positive family history for cardiovascular disease [19].

Therefore, we postulate that the similar frequency of those risk factors in the TTS and STEMI cohorts is due to their impact on the pathogenesis of those diseases. However, we found a higher proportion of smokers in the STEMI cohort than in the TTS cohort although the frequency of smokers in the TTS cohort was also relatively high.

Another interesting finding of our study is that the frequency of hyperlipidemia is significantly lower in the TTS cohort than in the STEMI cohort. Hyperlipidemia is associated with a higher risk of development of macrovascular diseases, but notably not causally associated with a higher risk of microvascular diseases [20]. In our opinion, this could explain the lower frequency of patients with hyperlipidemia in the TTS cohort although the difference in gender distribution between the TTS and STEMI cohort is also a plausible cause for the discrepancy of the frequency of hyperlipidemia.

### Outcome

We observed an in-hospital complication rate of 20% in TTS patients using the combined endpoint. A similarly high rate of in-hospital complications in TTS patients in Austria has also been described by Zweiker et al. providing further evidence that TTS should not be considered a benign disease [21]. Looking at the impact of pre-existing diseases on the short-term outcome of TTS patients, only pre-existing neurological diseases showed a trend towards adverse outcome in multivariate analysis, although no statistical significance could be shown (*p* = 0.07). This may be due to our small sample size, because a previous study with a larger sample size showed a significant association between neurological diseases and in-hospital mortality in TTS patients [22].

Evidence regarding the influence of cardiovascular risk factors on the short-term outcome of TTS is still scarce. An association of hyperlipidemia with better in-hospital outcome in TTS patients has previously been described [23]. However, although we observed a trend in univariate analysis, there was no statistically significant impact of hyperlipidemia on the short-term outcome of TTS patients in multivariate analysis. The authors postulated that an anti-inflammatory effect of statins might positively influence the outcome of patients with TTS and hyperlipidemia [23]; however, other studies showed no significant impact of statins on the short-term outcome or recurrence of TTS patients [24, 25]. Our data suggest that hyperlipidemia does not have an association with better short-term outcome, although we think further studies are necessary to evaluate the effects of hyperlipidemia in TTS patients.

Additionally, our study showed no significant influence of type II diabetes mellitus on the short-term outcome of TTS, which is in line with findings from a previous study [26].

Smoking has been associated with higher 30-day and 5‑year mortality in a previous study [27]; however, this was not evaluated in our study. Regarding only in-hospital complications we did not find an association between smoking and adverse events in TTS patients. Interestingly, in a study by Li et al. positive family history for cardiovascular disease was associated with favorable outcomes in TTS patients [28] which is contrary to findings of our study.

Furthermore, we found no significant association of hypertension or chronic kidney disease with the short-term outcome of TTS patients, which, to the best of our knowledge, has not been evaluated yet. However, a study by Pogran et al. found an association of chronic kidney disease with long-term mortality in TTS patients, which was not evaluated in our study [29].

Physical trigger factors were the only significant predictors for adverse short-term outcome in our study as also described by previous studies [5]. Increased catecholamine release due to acute physical or emotional stress is another hypothesis for the pathophysiology of TTS [2] and physical triggers are suspected to lead to a higher catecholamine surge which might explain the worse prognosis of those patients. However, in 12–31% of the cases no specific trigger for the genesis of TTS was identified [30]. This leads us to postulate that the level of catecholamine release might impact the outcome of TTS patients but may not be the (only) cause for the pathogenesis of TTS.

Although our data suggest that some cardiovascular risk factors play a role in the pathophysiology of TTS, they do not seem to have an impact on the short-term outcome of TTS patients. We consider TTS has a multifactorial genesis and hypothesize that TTS occurs in patients with coronary microvascular dysfunction; however, the outcome of TTS is determined by other factors such as the type of trigger, co-existing neurological disorders, and levels of catecholamines.

Further studies are required to evaluate the influence of coronary microvascular dysfunction and cardiovascular risk factors on the pathogenesis and outcome of TTS.

### Limitations

There are several limitations to our study that need to be taken into consideration. First, this is an observational study and cannot demonstrate causality as selection bias and residual confounding cannot be excluded. Second, our sample size is relatively small, however, given the rare nature of the disease it is still a reasonable sample size. Third, data were retrospectively collected and STEMI patients were only included from 2019 which may impact the quality of our data. Fourth, we included TTS data from a wide observational period and confounding factors such as differences in treatment strategies over time cannot be excluded. However overall, we have pursued the data collection and consistency checks according to high standards and we are convinced that the data are accurate.

## Conclusion

Psychiatric, neurological, and pulmonary pre-existing diseases are more common in TTS than in STEMI patients. The frequency of hypertension, type II diabetes mellitus, chronic kidney disease and positive family history for cardiovascular disease of TTS patients is similar to STEMI patients. Interestingly, pre-existing diseases and conventional cardiovascular risk factors do not seem to have any impact on the short-term outcome of TTS patients.
